# The Structure of Tumor Endothelial Marker 8 (TEM8) Extracellular Domain and Implications for Its Receptor Function for Recognizing Anthrax Toxin

**DOI:** 10.1371/journal.pone.0011203

**Published:** 2010-06-18

**Authors:** Sheng Fu, Xiaohang Tong, Chenguang Cai, Ying Zhao, Yang Wu, Yuanyuan Li, Junjie Xu, Xuejun C. Zhang, Long Xu, Wei Chen, Zihe Rao

**Affiliations:** 1 National Laboratory of Biomacromolecules, Institute of Biophysics, Chinese Academy of Sciences, Beijing, China; 2 Laboratory of Structural Biology, Tsinghua University, Beijing, China; 3 State Key Laboratory of Pathogen and Biosecurity, Beijing Institute of Microbiology and Epidemiology, Beijing, China; 4 Laboratory of Protein Engineering, Beijing Institute of Biotechnology, Beijing, China; 5 College of Sciences and Tianjin State Laboratory of Protein Sciences, Nankai University, Tianjin, China; University of Washington, United States of America

## Abstract

Anthrax toxin, which is released from the Gram-positive bacterium *Bacillus anthracis*, is composed of three proteins: protective antigen (PA), lethal factor (LF), and edema factor (EF). PA binds a receptor on the surface of the target cell and further assembles into a homo-heptameric pore through which EF and LF translocate into the cytosol. Two distinct cellular receptors for anthrax toxin, TEM8/ANTXR1 and CMG2/ANTXR2, have been identified, and it is known that their extracellular domains bind PA with low and high affinities, respectively. Here, we report the crystal structure of the TEM8 extracellular vWA domain at 1.7 Å resolution. The overall structure has a typical integrin fold and is similar to that of the previously published CMG2 structure. In addition, using structure-based mutagenesis, we demonstrate that the putative interface region of TEM8 with PA (consisting of residues 56, 57, and 154–160) is responsible for the PA-binding affinity differences between the two receptors. In particular, Leu56 was shown to be a key factor for the lower affinity of TEM8 towards PA compared with CMG2. Because of its high affinity for PA and low expression in normal tissues, an isolated extracellular vWA domain of the L56A TEM8 variant may serve as a potent antitoxin and a potential therapeutic treatment for anthrax infection. Moreover, as TEM8 is often over-expressed in tumor cells, our TEM8 crystal structure may provide new insights into how to design PA mutants that preferentially target tumor cells.

## Introduction

Anthrax is a lethal infectious disease caused by *Bacillus anthracis*, a Gram-positive, spore-forming, rod-shaped bacterium that secretes three toxin proteins: lethal factor (LF), edema factor (EF), and protective antigen (PA) [1]. They are collectively called anthrax toxin, and PA is responsible for interaction with receptors on target cell surfaces [2].

Anthrax toxin enters target cells through a multi-step mechanism. First, full-length PA (PA_83_, 83 kDa) binds cell surface receptors and is cleaved by cellular furin-like enzymes [3], [4]. The remaining activated PA protein (PA_63_, 63 kDa) then oligomerizes into a heptameric structure known as a prepore and interacts with EF and/or LF, which are located between two adjacent PA monomers [5], [6], [7], [8]. The entire receptor–toxin complex is then transported into low-pH endosomes via endocytosis [9]. Pore formation across the endosomal membrane is triggered by increasing acidity, which induces a pivotal conformational rearrangement of the prepore assembly [2], [10]. Thus, understanding the PA-receptor interaction is critical for anthrax toxicity prevention and other potential therapeutic applications.

By using a genetic approach, two PA cell surface receptors have been identified: TEM8/ANTXR1 (tumor endothelial marker 8/anthrax toxin receptor 1) and CMG2/ANTXR2 (capillary morphogenesis protein 2/anthrax toxin receptor 2) [11], [12]. CMG2 is the major receptor mediating lethality of anthrax toxin *in vivo* [13]. TEM8 and CMG2 are type I transmembrane proteins with three domains: an N-terminal, extracellular von Willebrand factor type A domain (vWA domain), a single transmembrane spanning domain, and a C-terminal cytosolic domain [11], [12]. Their vWA domains share approximately 55% sequence identity. Compared to the wide distribution of CMG2 in normal adult tissues (*e.g.*, lung, brain, kidney, and muscle), TEM8 is only weakly detected in these tissues but abundant in tumor endothelial cells and the vasculature of developing embryos [11], [14], [15]. This is one of the reasons that CMG2 plays a more important role in anthrax toxin transportation into cells than TEM8. The physiological ligands of these two receptors, as well as their cellular functions, remain elusive. Interestingly, the tissue distribution differences between these proteins allow the specific targeting of tumor cells using the PA–receptor system.

Moreover, the PA-binding affinities of TEM8 and CMG2 are strikingly different. CMG2 was reported to have approximately 1000-fold higher affinity for PA (K_d_ = 170 and 780 pM for the Mg^2+^- and Ca^2+^-bound complexes, respectively) than TEM8 (K_d_ = 1.1 µM and 130 nM for the Mg^2+^- and Ca^2+^-bound complexes, respectively) [16], [17], the latter of which is about the average level of integrin-ligand interaction [18]. Structural details and mutation analysis of TEM8 will be necessary to explain the huge difference in PA-binding affinity between TEM8 and CMG2. Meanwhile, PA mutants that can selectively bind with either TEM8 or CMG2 have been designed and tested, and they are potential therapeutic agents for cancer treatment [19].

The MIDAS (metal ion-dependent adhesion site) motif in vWA domains can exist in either of two conformations, much like the integrin I domain: closed (low-affinity ligand binding state) and open (high-affinity ligand binding state) [20]. In a previously reported CMG2 structure, the metal ion-coordinating residues of CMG2 adopted the open state with an acetate molecule as a mimic ligand, and such an open form is believed to attribute to the higher affinity of CMG2 towards PA [10], [14], [21], [22]. Results from mutational analysis suggested that TEM8 also adopts an open conformation [23]. However, such a notion remains to be proved experimentally.

The pH threshold for conversion of the PA prepore to the pore and toxin translocation is also receptor-specific. The pH required for CMG2-associated toxin pore formation (pH 5.0) is lower than that of TEM8 (pH 6.0) [24]. Interestingly, the CMG2 Y119H variant with a mutation in the ligand-binding pocket further lowers the pH threshold [25], [26], and CMG2-mediated intoxication is blocked by ammonium chloride (NH_4_Cl) treatment, which raises the endosomal pH [24]. A model of CMG2-associated toxin prepore-to-pore conversion has been proposed in which the receptor restrains the membrane insertion loop (β2–β3, residues 285–340) of PA domain 2 until protonation of PA and/or CMG2 residues loosen this interaction to allow PA domain 2 to form an extended β-barrel pore. However, determining whether this model is suitable for TEM8-mediated intoxication and which key TEM8 residues are responsible for the different pH thresholds requires additional TEM8 structural information, particularly concerning its vWA domain.

Here we report the high resolution structure of the TEM8 vWA domain. We found that the vWA domain contains a chelated Mg^2+^ ion and a bound pseudo-ligand (*i.e.*, an acetate ion from the crystallization buffer) in its MIDAS site. Based on structural analysis, we discuss a probable structural explanation for the difference between TEM8- and CMG2-mediated toxin interaction and pore formation. We also carried out a systematic mutational analysis of TEM8 using cell protection assays, surface plasmon resonance (SPR), and pH-dependent SDS-PAGE to verify our hypothesis.

## Results

### Overall structure of the extracellular domain of TEM8

We obtained only one qualified crystal after screening more than 200 crystals in two crystallization conditions. The crystal belongs to the P1 space group and diffracted up to 1.7 Å resolution. The crystal structure was solved using the molecular replacement method from synchrotron data (PF, Japan). The model was built from residues Ala38 to Cys220; Eight residues (MSHHHHHH) of N-terminal affinity tag were not modeled due to poor electron density and the following two residues (SM) linked to the target protein can be determined in the electron density map and refined as residue 36 and 37 in the final model. The structure model was refined to a final R-factor of 0.194 and R-free of 0.232 (Supplemental Data Table S1).

**Figure 1:**
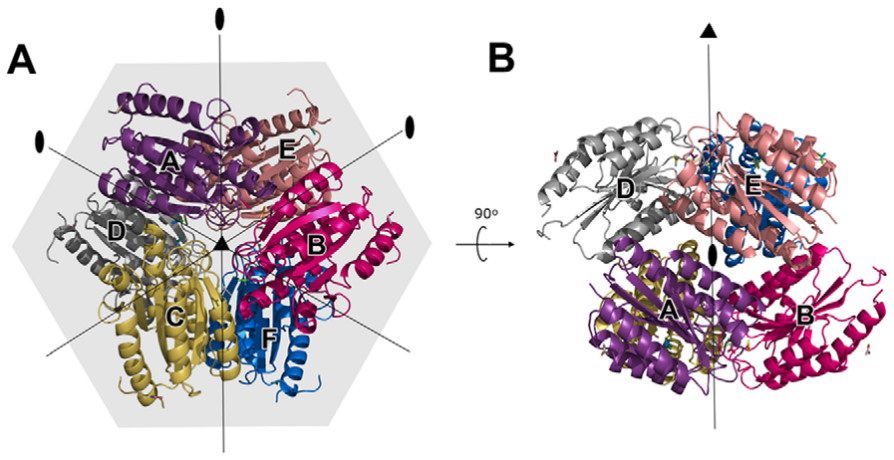
TEM8 vWA domain hexamer in the crystal cell. Protein molecules are shown in ribbon presentation. Six TEM8 vWA domain molecules (labeled A–F) form two trimers, which further assemble into a ball shape in the asymmetric unit. Each TEM8 molecule is shaded with a unique color. The dimensions of the ball are ∼82×82×66 Å.

Our crystal structure contained six TEM8 vWA molecules (labeled as A, B, C, D, E, and F) in the asymmetric unit (also unit cell) (Figure 1). Three monomers were related by a non-crystallographic three-fold symmetry to form a trimer, and two of such trimers (A-B-C and D-E-F) formed a hexamer with an overall ball-like shape through a two-fold axis perpendicular to the three-fold axis (Figure 1A, 1B). The dimensions of the ball are ∼82×82×66 Å. In this hexamer, the MIDAS ligand binding area from each monomer was blocked, which would definitely interrupt the interaction between TEM8 and PA (Supplemental Data Figure S1). This indicates that such a hexamer is an inactive oligomeric form. Moreover, TEM8 was determined to exist as a monomer in solution by analytical ultracentrifuge (Supplemental Data Figure S2) and gel filtration. Thus, the observed TEM8 hexamer is most likely an artifact of crystal packing.

**Figure 2:**
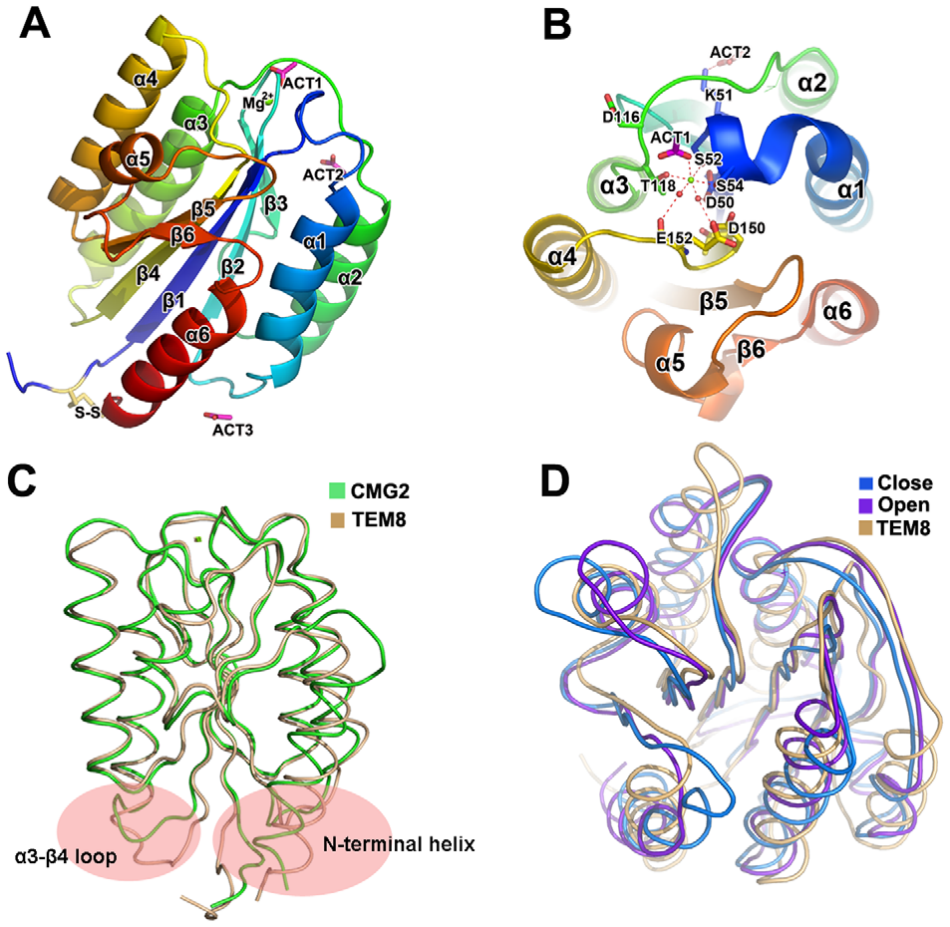
Overall structure of the TEM8 vWA domain and its open conformation. (**A**) TEM8 vWA domain structure (side view). Five parallel β strands (β_1_, β_2,_ β_4_, β_5_, and β_6_) and one short anti-parallel β-strand (β_3_) form a central sheet that is surrounded by six α-helices (α_1_–α_6_). This structure contains a chelated Mg^2+^ ion (light green sphere) in the MIDAS site, with a bound pseudo-ligand contributed by an acetate ion (ACT1, purple). Two additional acetate ions (ACT2 and ACT3, purple) are also observed in the structure. (**B**) TEM8 MIDAS site (top view). Ser52 and Ser54 in helix α1 (blue) and Thr118 in α3 (green) form direct bonds that coordinate the Mg^2+^ ion, while Asp50 in α1 (blue) and Glu152 and Asp150 in the α4–β4 loop (yellow) form water-mediated hydrogen-bonds to the metal ion. The rest of the MIDAS coordination site is occupied by a mimic ligand (*i.e.*, the acetate ion, ACT1, purple). (**C**) TEM8 vWA domain structure (colored clay) was superimposed onto the CMG2-S38 vWA domain structure (colored green, PDB ID 1SHU). The most distinct sites are highlighted by pink ovals. (**D**) The superposition (top view) between the TEM8 vWA domain structure (clay), domain I structure of Integrin CR3 in the open conformation (purple, 1IDO) and CR3 in the closed conformation (blue, 1JLM). The TEM8 vWA structure is much closer to the open conformation.

Consistent with their sequence homology, the structure of the TEM8 extracellular domain is very similar to that of CMG2 and the integrin A domain [21], [27]. It adopts a classical α/β open sheet fold that has also been called the dinucleotide-binding fold, Rossmann fold, or doubly wound fold [21]. Five parallel β-strands (*i.e.*, β1, residues 42–50; β2, 77–85; β4, 73–79; β5, 173–179; and β6, 196–199) and one short antiparallel β strand (β3, 89–96) form a central β-sheet. The hydrophobic residues of the β-sheet form a hydrophobic core on each side, surrounded by six amphipathic α-helices (α1, 53–72; α2, 99–110; α3, 120–135; α4, 141–149; α5, 155–170; and α6, 200–217) (Figure 2A). It should be noted that three acetate ions were found in each of the six TEM8 molecules and were well defined in the electron density map. One of them appeared to correspond to an analogous ion in the CMG2 structure, which acted as a mimic ligand of the side chain of D683 in PA and occupied the MIDAS coordination site (Figure 2B). The existence of the other two acetate ions may be incidental to our crystal form due to the 0.2 M ammonium acetate included in the crystallization buffer.

### An open conformation of the TEM8 vWA domain

The extracellular domain of TEM8 shares a common topology with a wide variety of intracellular enzymes and cell adhesion molecules. Not surprisingly, CMG2 is the most similar structure to TEM8, according to the 3D structure similarity search engine DALI [28]. Two crystal structures of CMG2 have been reported (PDB ID 1SHU and 1SHT), and the structural superpositioning of the TEM8 vWA domain with CMG2 yielded a root mean square deviation (rmsd) of 1.2 Å for 175 common Cα atoms in CMG2-S38 (residues 38–218; 1SHU) and 1.6 Å for 168 common Cα atoms in CMG2-R40 (residues 40–217; 1SHT). The main quaternary structure differences between TEM8 and CMG2 occurred in the α3–β4 loop and helix α6. First, the TEM8 α3–β4 loop moved 10 Å compared with CMG2, and the sequence of the α3–β4 loop varied between TEM8 and CMG2. Second, while the N-termini of α6 in the two structures were located at the same position, this conserved helix extended in different directions (Figure 2C).

The TEM8 vWA domain structure also showed high similarity with domain I of Integrin CR3 in the open conformation (2.1 Å rmsd for 155 Cα atoms, PDB ID 1IDO [27]), CR3 in the closed conformation (2.2 Å for 156 Cα atoms, 1JLM [27]), Complement Factor B (2.0 Å for 162 Cα atoms, 1RRK [29]), complement C2 (2.1 Å for 164 Cα atoms, 2ODP [30]), and Von Willebrand Factor (2.5 Å for 172 Cα atoms, 3GXB [31]), although they have low sequence identity (18–26%). The integrin I domain has two conformations, open and closed (Figure 2D), and the conversion between these two conformations usually plays an important biological function, representing the active and inactive states, respectively [18], [20], [27]. In the open conformation, two serines and a threonine residue tightly bind to the metal ion, and two water molecules also bind directly to the metal ion, similar to that described above for TEM8. In contrast, in the closed conformation, the metal ion shifts, and only the threonine can indirectly contact the ion via a water molecule. Taken together, the TEM8 extracellular domain contained a conserved Mg^2+^-coordinated MIDAS motif that assumed an integrin-like open conformation.

### The PA-binding interface of two receptors

**Figure 3:**
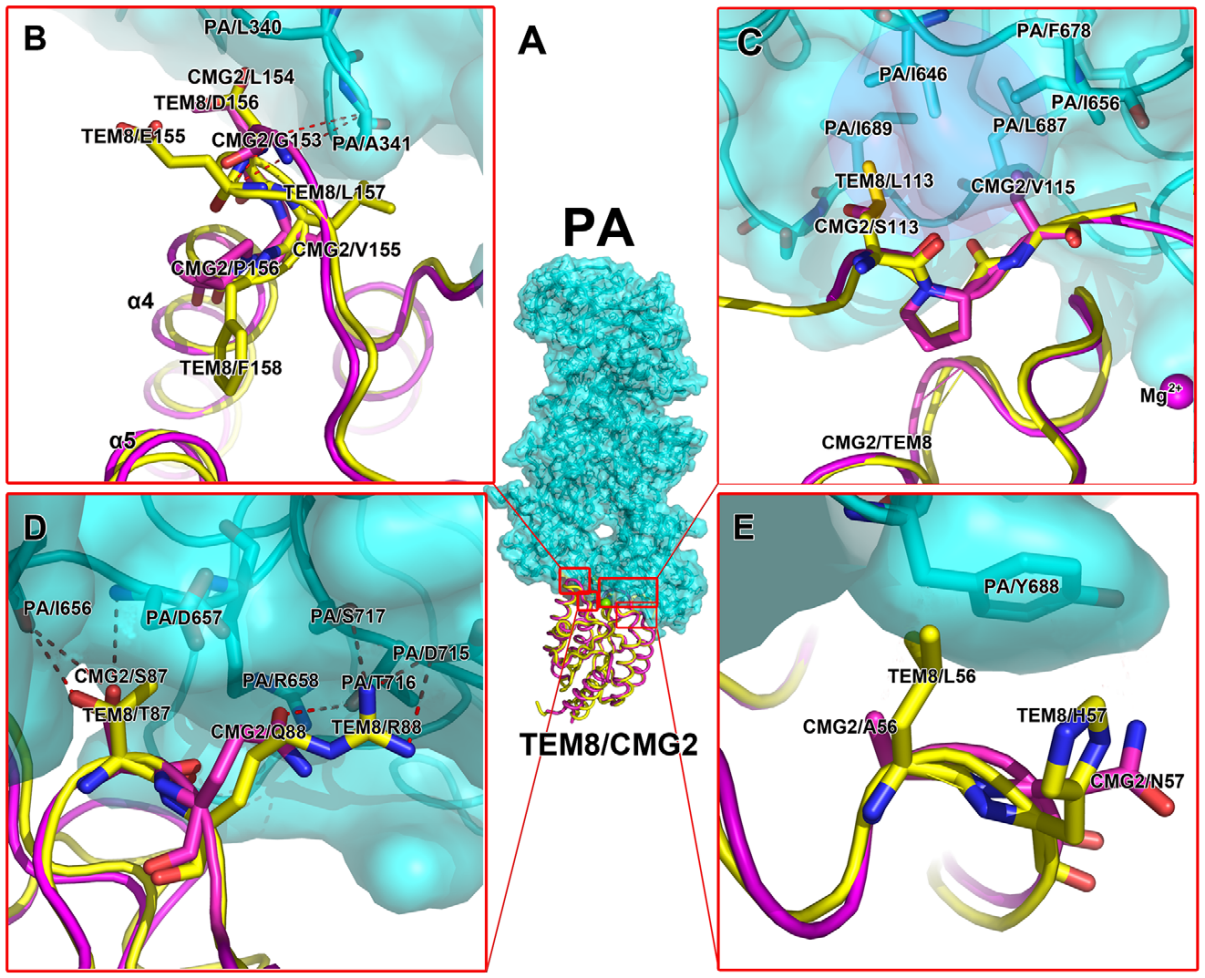
TEM8-PA_63_ complex model and details of their interaction. (**A**) A model of PA_63_ and TEM8 vWA domain complex (PA_63_, cyan; TEM8 vWA domain, yellow; Mg^2+^, green; and acetate ion, purple). The TEM8 vWA domain is superimposed onto the CMG2 vWA domain according to the structure of the CMG2-PA complex (PDB ID 1T6B). (**B**–**E**) Comparison of the TEM8-PA_63_ binding surface and CMG2-PA_63_ binding surface. According to our TEM8-PA_63_ complex model, there are four TEM8 regions anticipated to interact with PA_63_. Here, we show detailed structural differences between the TEM8-PA_63_ binding surface and the CMG2-PA_63_ binding surface (TEM8, yellow; CMG2, purple; and PA_63_, cyan). The molecular surface of PA (cyan, semi transparent) is also included. (**B**) Part 4 (residues 153–158), located in the β3–β4 loop interacts, with Leu340 and Ala341 in domain 4 of PA_63_. (**C**) Part 3 (residues 113 and 115), located in the α2–α3 loop, interacts with a hydrophobic cleft comprised of Leu687, Ile689, Ile646, Phe678, and Ile656 of PA_63_. The picture is reverse with box in A (**D**) Part 2 (residues 87 and 88), located in the β2–β3 loop, interacts with Asp657, Arg658, Asp714, and Thr715 of PA_63_. (**E**) Part 1 (residues 56 and 57), located in helix α1, interacts with Tyr688 in domain 2 of PA_63_.

Although TEM8 and CMG2 share high sequence identity, they widely differ in binding affinities for PA and in pH thresholds for forming SDS-resistant pores. The previously reported CMG2-PA complex structure depicts extensive contact between CMG2 and PA domains 2 and 4 [22]. CMG2 has a much larger contact surface (∼2000 Å^2^) than a typical α-integrin–ligand (∼1300 Å^2^), and it was believed that this larger surface is responsible for the higher affinity of CMG2-PA binding [22], [32]. However, we found that the buried surface area between TEM8 and PA is very similar to that of CMG2, as calculated by our TEM8-PA complex structure model. According to the CMG2-PA complex and TEM8 structures, we superpose the TEM8 structure to CMG2-PA with COOT and generate the TEM8-PA complex (Figure 3A). Thus, a direct relationship between the buried surface area and affinity does not hold. In order to investigate the structural basis of these differences, we further compared the ligand binding sites of TEM8 and CMG2.

The Mg^2+^ ion from the MIDAS site of CMG2 is directly involved in PA-binding. Based on the sequence alignment of the two receptors, there is a two residues width gap (from 135 to 136 of CMG2) between the TEM8 and CMG2 vWA domains. Interestingly, most of those interface residues in CMG2 are not conserved in the corresponding TEM8 sites (*i.e.*, residues 56, 57, 87, 88, 113, 115, 117, 125, 154, 155, and 156 in TEM8). We can divide these non-conserved residues into four regions in TEM8: Part 1 (residues 56 and 57) is located in helix α1 (the corresponding part in CMG2 interacts with domain 2 of PA); Part 2 (residues 87 and 88) is located in the β2–β3 loop; Part 3 (residues 113–117, Tyr119, His121, Glu122, and Glu125) is located in the α2–α3 loop; and Part 4 (residues 152–156 and Tyr158) is located in the β3–β4 loop (Figure 3). Together, the CMG2 Part 2–4 counterparts interact with domain 4 of PA. We hypothesized that these non-conserved residues in the PA-binding interface are responsible for the striking differences in PA binding affinity, receptor-specific pH thresholds for pore formation, and even translocation of the toxin.

### Key residues resulting in the difference of binding affinity between TEM8 and CMG2

We designed a number of TEM8 single point mutants (*i.e.*, L56A, H57N, T87S, R88Q, D117E, H154D, E155G, D156L, L157V, F158P, K51A, Y119H, Y119R, E122A, and E122H) and multiple site mutants M1 (L56A and H57N) and M2 (L56A, H57N, H154D, D156L, L157V, and F158P). As stated in the Materials and Methods, the C177A mutation was also introduced in all of the above mutants in order to reduce aggregation of the recombinant protein. Unfortunately, some of the mutants were still insoluble in the *E. coli* expression system and were excluded from further studies. We purified the soluble mutants (*i.e.*, L56A, H57N, T87S, R88Q, D117E, H154D, L157V, F158P, K51A, Y119H, Y119R, E122A, E122H, M1, and M2) and indirectly tested their PA binding affinity using a J774 A.1 cell protection assay and the SPR method.

**Figure 4:**
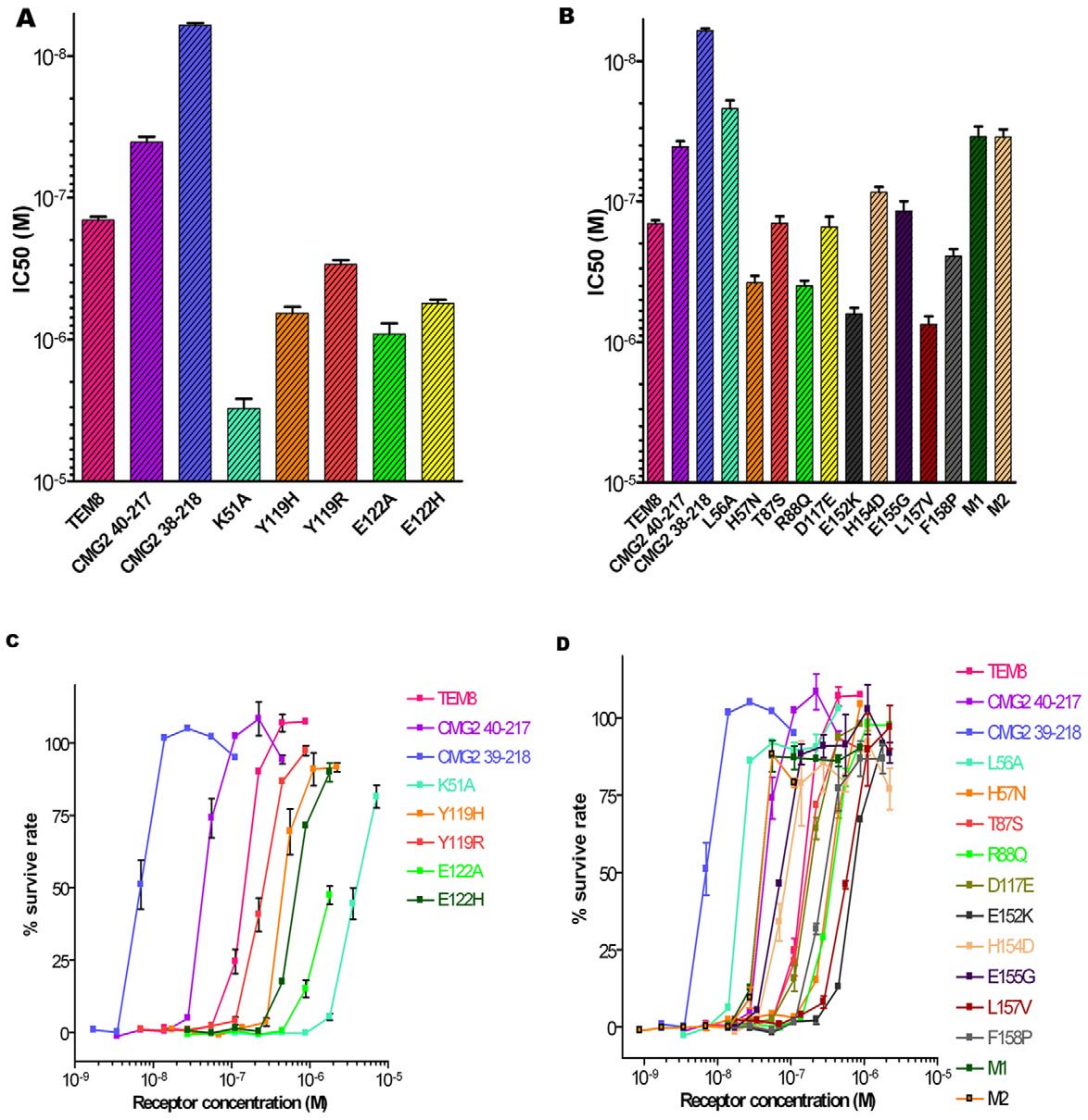
Inhibition ability of receptor variants (vWA domain) for protecting J774A-1 cells from PA intoxication. The survival rate is calculated using the equation:
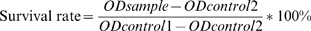
Control 1 is cells treated without either PA or receptors (mutants) and control 2 is cells treated with PA but without receptors. (**A**) The protective ability (IC_50_) of TEM8 variants that replace the original residue with Ala or contrary charged residue at the conserved sites in the binding interface between TEM8 and CMG2. (B) The protective ability (IC_50_) of TEM8 variants that replace the original residue with the corresponding residue in CMG2 at the non-conserved sites. Data points and error bar represent the mean ± SEM values for three independent experiments in (A) and (B). (C) Survive curves show the negative Log value of the IC_50_ by TEM8 variants/receptors, based on results showed by (A). In the same way, (D) is the corresponding curves of (B). Data points and error bar represent the mean ± SEM values for one representative experiment with duplicates in (C) and (D).

The cell protection results showed that TEM8 Part 1 mutants M1, M2, and especially L56A significantly increase the protective ability (up to 7-fold in terms of IC_50_) compared to WT TEM8. Their protective ability even exceeded the CMG2-R40 protein (*i.e.*, residues 40–217), in the absence of the only disulfide bond between Cys39 and Cys218, and was one-third as strong as the CMG2-S38 variant (*i.e.*, residues 38–218) containing the disulfide bond. In contrast, the H57N, R88Q, L157V, and F158P point mutants had decreased protective ability. Further, the Part 4 H154D mutation resulted in a mild improvement of the PA-binding ability, and Part 2 mutant T87S and Part 3 mutant D117E had almost the same protective effect as WT TEM8. These results indicated that TEM8 Parts 1 and 4 are likely to be the main contributors to the PA affinity differences between TEM8 and CMG2 (Figure 4).

Additionally, we used SPR to directly test whether the key residues identified in the cell protection assay have different PA binding abilities (Table 1). In particular, we tested the K51A, L56A, T87S, R88Q, M1, and M2 mutants. The results showed a clear correlation between cell protection ability and PA binding ability. For instance, the L56A mutant had the highest PA-binding affinity (K_D_ of 4.4 nM) among the mutants tested, which is close to the affinity of CMG2-R40 (2.4 nM). The M1 mutant also showed a PA binding affinity (5.3 nM) similar to that of CMG2. For comparison, the K_D_ value of TEM8 WT was 29.8 nM. We should mention that this WT TEM8 K_D_ we measured was 4-fold lower than previous reported (130 nM), but this difference is acceptable considering SPR system characteristics [17] and we make sure this result was from very rigorous repeatable experiments. Finally, the Part 2 T87S and R88Q mutants had K_D_ values of 49.7 nM and 167 nM, respectively, indicating that these Part 2 mutants have 2- to 5.5-fold lower PA binding affinity than the WT.

**Table 1:** The PA-binding kinetic ratio of CMG2^R40^, TEM8 and its mutants based on SPR data.

Mutants	k_a_ (M^−1^s^−1^)	k_d_ (s^−1^)	K_D_ (M^a^)	replicates
**TEM8**	5.46E+03±1.56E+03	1.40E−04±5.76E−05	2.98E−08±8.88E−09	4
**CMG2^R40^**	4.53E+04±3.42E+04	1.05E−04±7.67E−05	2.43E−09±1.40E−10	2
**K51A**	9.36E+01±2.14E+01	5.27E−03±5.20E−04	5.81E−05±7.80E−06	2
**L56A**	1.49E+04±2.12E+02	6.60E−05±9.19E−06	4.44E−09±5.37E−10	2
**T87S**	6.11E+03±4.09E+03	2.59E−04±1.44E−04	4.97E−08±7.30E−09	2
**R88Q**	3.73E+03±3.50E+02	6.21E−04±8.00E−06	1.67E−07±4.00E−09	2
**M1**	8.66E+03±1.95E+03	4.46E−05±6.9E−06	5.25E−09±3.75E−10	2
**M2**	4.83E+03±3.65E+02	5.79E−05±5.55E−06	1.20E−08±2.50E−10	2

a The equilibrium dissociation constant was calculated from kinetic measurements of the association and dissociation rate constants according to *K_D_ = k_d_/k_a_*.

b TEM8: TEM8 residues 38–220 with a C177A mutation as mentioned in materials and methods; CMG2^R40^: CMG2 residues 40–217; M1 contains L56A and H57N mutations; M2 contains L56A, H57N, H154D, E155G, D156L, L157V, and F158P mutations.

**Figure 5:**
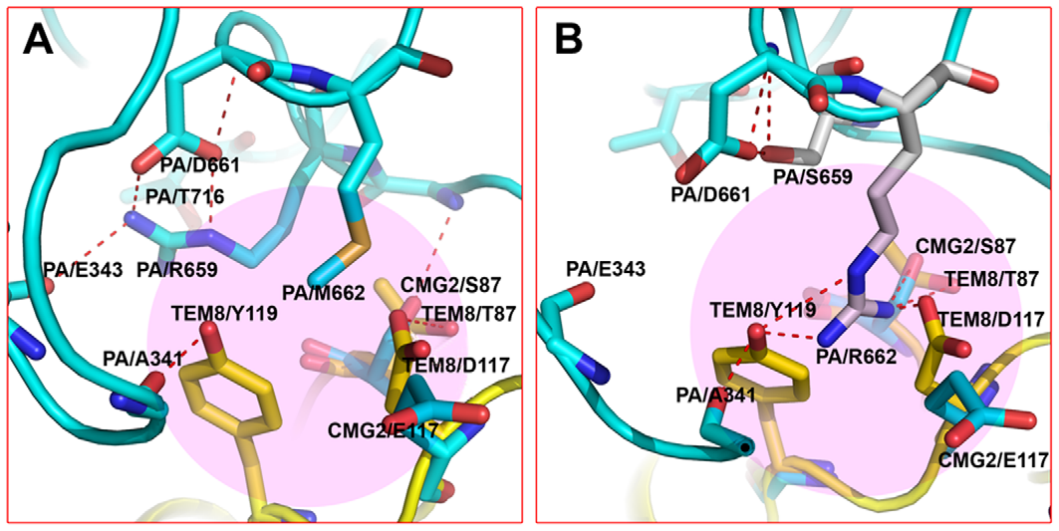
Comparison of PA-CMG2 and PA M662R/R659S-CMG2. (**A**) PA-CMG2 complex (light blue, PDB ID 1T6B). The TEM8 vWA domain, colored yellow, is superimposed onto the CMG2 vWA domain of the complex. (**B**) Model of the PA mutant (M662R/R659S) complexed with CMG2, according to the structure of the PA-CMG2 complex. The TEM8 vWA domain was superimposed onto the CMG2 vWA domain. The mutated residue is colored grey.

We also tested the effects of alanine substitution mutations of some conserved residues (*e.g.*, Lys51, Tyr119, and Glu122) in TEM8. All of these mutants, especially K51A, reduced the protective effect (K_D_ decreased by 22-fold) (Figure 4, Table 1). The conserved Lys51 residue plays an important role in stabilizing the complex by making strong hydrogen bonds to PA Glu654, as shown in the CMG2-PA complex [22]. Moreover, several groups have independently discovered that CMG2 Tyr119 plays a key regulatory role in acid pH-dependent pore formation [25], [26]. Our cell protection data indicated that TEM8 Tyr119 also plays an important role in protective ability and PA binding affinity (Figure 4). According to our TEM8-PA complex model, TEM8 Tyr119 inserts into a planar cleft between domains 2 and 4 of PA and forms a hydrogen bond with the backbone carbonyl oxygen of PA Ala341(Figure 5A). In addition, TEM8 Glu122 forms a salt bridge with PA Arg344. Thus, all of these conserved residues play important roles in PA binding.

## Discussion

### A possible explanation of mutation results based on the PA-TEM8 complex model

In order to explain why the key residues of the two receptors identified above play important roles in the difference of PA-binding ability, we superimposed the TEM8 structure onto the CMG2 molecule in the CMG2-PA complex structure, thus creating a putative TEM8-PA complex model (Figure 3A). The TEM8 L56A mutant stands out among all of the tested mutations. It showed a sharp increase of protective ability and PA binding affinity. This indicates that TEM8 Leu56 is an important determinant of the difference between TEM8 and CMG2. This may be explained by the structural observation that the side chain of Leu56 clashes with a PA Tyr688 in our putative PA-TEM8 complex model (Figure 3E). In contrast, the corresponding CMG2 Ala56 has a short side chain and hence allows the CMG2 molecule (residues 113 and 115) to contact a hydrophobic area of PA (residues 687, 689, 646, and 652). In addition, according to the data from the cell protection and SPR assays, both M1 and M2 mutants, which contain the L56A mutation, had a striking increase in protective ability and PA binding affinity, further supporting the important role of position 56 in determining PA-binding affinity.

Another readily noticeable 3D structural distinction can be observed at residues 154–160 (HEDLFFY) in TEM8; the corresponding residues in CMG2 are 152–158 (DGLVPSY) (Figure 3B). In the PA-CMG2 complex structure (PDB ID 1T6B), Gly153, Leu154, and Val155 residues participate in hydrophobic contacts with the side chains of PA residues Leu340 and Ala341. Therefore, substitution of Gly153 and Leu154 (in CMG2) with Glu155 and Asp156 (in TEM8) would disturb this hydrophobic interaction. In fact, an inspection of the TEM8-PA complex model suggested that such mutations induced a dramatic change of the main chain (a 3 Å shift away from PA) (Figure 3B), which would result in a loss of both the hydrophobic and Van der Waals interactions. Previously, Young, Collier, and coworkers identified that CMG2 G153 and L154 are key residues related to the difference in PA binding ability and pH threshold [26]. It was also hypothesized that mutations of Glu155 and Asp156 in TEM8 Part 4 would significantly affect PA binding affinity because there is a significant position shift in the host loop between TEM8 and CMG2. Unfortunately, the TEM8 D156L mutant (a substitution to the corresponding CMG2 residue) was not expressed in a soluble form in *E. coli*. We suspect that mutations in this loop may have negative effects on overall stability. Furthermore, TEM8 Leu157 is located at a position similar to CMG2 Val155 (Figure 3B) but appears to have a more extensive interaction with a PA hydrophobic surface. Consistent with this observation, TEM8 L157V showed a lower PA binding (Figure 4). In our TEM8 crystal structure, Phe158 is inserted into the hydrophobic core of TEM8, and this insertion enlarges the distance between the α4 and α5 helices (Figure 3B). However, this change did not obviously affect the binding ability (Figure 4B).

The other two parts of the receptor interface with PA, Part 2 (87–88) and especially Part 3 (113–122), bury a large amount of surface area upon PA binding. The α2–α3 loop of TEM8 forms a hydrophobic ridge that inserts into a groove formed by a β-sandwich of the immunoglobulin-like fold of PA domain 4. Non-intuitively, some of the TEM8 single mutations in Part 2 and Part 3 showed no significant effect on protective ability and PA binding. Regardless, previous research also showed that the CMG2 S113L and V115G mutants (equivalent to positions 113 and 115 in TEM8) cause no change in protective ability [33]. Hence, we sought to find some structural explanation for this structural tolerance (Figure 3C, 3D). For example, in the TEM8 structure Thr87 is hydrogen-bonded to the PA Ile656 backbone, and this interaction is unperturbed in our T87S mutation. In contrast, the R88Q mutant indeed reduced the protective ability (Figure 4 and Table 1). The terminal amino nitrogen group, NH1, of the Arg88 side chain forms a strong hydrogen bond with PA Ser717, and the other amino nitrogen group, NH2, of the Arg88 side chain forms a salt bridge with PA Asp715. In the mutant, these two bonds were replaced by a single hydrogen bond between Gln88 and PA Thr716. This structural change weakens the interaction network and causes a decrease in binding affinity. Therefore, TEM8 Parts 2 and 3 are likely to be essential in PA-binding, similar to the equivalent parts in CMG2.

### The relationship between binding affinity and the pH threshold of pore formation

**Figure 6:**
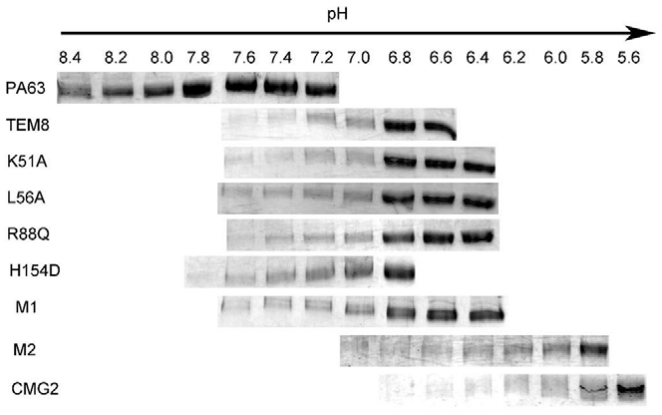
pH threshold of pore formation of TEM8 variants. Formation of SDS-PAGE resistant oligomers is assayed at different pH values in solution with the vWA domain of the WT receptors and TEM8 mutants. Experiments have been repeated at least twice and the results were identical.

Another characteristic difference between TEM8 and CMG2 is that the pH threshold for conversion of a PA prepore to pore is altered by one full pH unit (TEM8, pH 6.0 *in vivo* and pH 6.8–7.1 *in vitro*; and CMG2, pH 5.0 *in vivo* and pH 5.7–5.8 *in vitro*) [24], [26]. In our *in vitro* system, when PA was bound to TEM8, formation of the SDS-PAGE resistant oligomer occurred at pH 6.8, and when bound to CMG2, its formation occurred at pH 5.6. In a previous report, Gly153 and Leu154, which contact PA domain 2, and residue Gln88, which contacts PA domain 4 (Glu155, Asp156, Arg88 in TEM8), are major determinants of the lower pH threshold requirement associated with CMG2 [26]. Correspondingly, Six TEM8 mutants (*i.e.*, K51A, L56A, R88Q, H154D, M1, and M2) were analyzed. M2 (154–158) showed a striking change in the pH threshold of pore formation, with a value as approaching to that of CMG2. Meanwhile, all single point mutations did not cause a significant change in the pH threshold of pore formation (Figure 6). We sought to determine whether there was a correlative relationship between the binding affinity and pH thresholds of pore formation in the two receptors. Interestingly, the K51A, L56A, and M1 mutants that significantly altered the binding affinity did not cause a significant change in the pH threshold of pore formation. Thus, our data do not support a simple, linear correlation between binding affinity and the pH threshold of pore formation in the relationship between TEM8 and PA.

### TEM8 L56A can be considered an anti-toxin drug candidate

It was reported that soluble CMG2 and TEM8 extracellular domains are antitoxins that can block intoxication of CHO-K1 cells by PA and LF [11], [12], [17]. Recently, a neutralizing monoclonal antibody against the PA of *B. anthracis* was developed, which can directly interact with the toxin and inactivate it [34], [35]. However, some *B. anthracis* strains can express functional but antigenically altered forms of PA, and such strains may elude treatment with such anti-PA antibodies. Thus, soluble receptor decoys are important in assisting antibody-based therapies [17]. However, the soluble WT TEM8 vWA domain is not as effective as its counterpart from CMG2 due to the lower PA-TEM8 binding affinity. Indeed, to protect cultured CHO-K1 cells against intoxication by 50% with soluble receptors, 200-fold more TEM8 is needed than CMG2 [17]. In the current study, the TEM8 L56A mutant was tested for its ability to block intoxication and was found to be very similar to CMG2. The IC_50_ of this TEM8 variant was estimated to be 21.9 nM, whereas the IC_50_ of WT TEM8 was 143.2 nM. For comparison, the IC_50_ of soluble CMG2 with and without the Cys39–Cys219 disulfide bond were estimated to be 6.1 nM and 40.6 nM, respectively (Figure 4). Considering the low expression of TEM8 in normal tissues, the TEM8 L56A mutant may incur less potential side-effects compared to the widely expressed CMG2, thus it may become a safer and more promising antitoxin than soluble CMG2.

### A possible mechanism of selective interaction between modified PA and receptors

TEM8 is difficult to detect in normal tissues but abundant in tumor cells. This presents a possibility of targeting drugs to tumor cells using a PA-TEM8-based system. Leppla and coworkers used phage display to select PA variants that preferentially bind to TEM8 over CMG2, in order to target tumor cells with modified anthrax toxin PA [19]. One of their candidates, the PA R659S/M662R protein, binds 10-fold more tightly to TEM8 than CMG2. A structural explanation can be deduced from a comparison of our PA-TEM8 model with the PA-CMG2 complex structure [21]. First, relative to WT PA, the M662R mutant may form two extra salt bridges with TEM8 (Figure 5B): one with Tyr119 (Tyr119 in CMG2) and the other with Asp117 (Glu117 in CMG2). However, the latter one does not appear at the PA-CMG2 interface (Figure 5B), thus giving TEM8 some extra binding advantage over CMG2. Second, in PA, the R659S mutation eliminates an intra-molecular hydrogen-bond with the Leu340 backbone. We speculate that such a mutation may enhance PA–receptor binding by giving the Leu340-residing loop more flexibility. Considering some residues in TEM8, such as Y119, E122 and L340, may not adopt the same configuration in the complex as shown the in free TEM8 crystal structure, so the above explanation may not reflect the real conformation change. However, this case still hints how the crystal structure of the TEM8 vWA domain serves as a structural model to lend support to manipulations of the TEM8-PA interaction and hence sheds light on potential antitumor therapies.

In this work, we determined the crystal structure of the TEM8 vWA domain at 1.7 Å resolution. The structure aids our understanding of how PA mediates anthrax toxin translocation into cells and sheds light on functional differences between the two anthrax receptors. The overall structure of TEM8 is quite similar to the previously reported CMG2 structure; yet there are numerous detailed structural differences. Among the four sequence regions that interact with PA in our putative TEM8-PA complex model, TEM8 Parts 1 and 4 in the PA binding interface were the main determinants for the large difference of PA binding affinity. Part 1 (residues 55 and 56) and Part 4 (residues 153 and 154) significantly affected binding affinity and partially influenced the pH threshold of pore formation. Moreover, we found that the TEM8 L56A mutant strikingly increased the PA binding affinity and hence can be used as a good decoy antitoxin. In addition, based on our PA-TEM8 complex model, we analyzed why the PA M662R/R659S mutant preferentially binds to TEM8 over CMG2, highlighting the possibility to create more effective PA mutants based on our TEM8 structure.

## Materials and Methods

### Protein expression and purification

The cDNA sequence encoding the human TEM8 (GenBank ID NP_115584.1) vWA domain (residues 38–220) was cloned into the pHAT2 vector (EMBL) for expression as an N-terminal His-tag fusion protein (The N-terminal affinity tag residues were MSHHHHHHSM). In order to reduce aggregation of the recombinant protein, we also introduced a C177A mutation in the wild type (WT) TEM8 clone and subsequent mutant variants. Recombinant C177A protein was expressed in the soluble fraction in *Escherichia coli* strain BL21 (DE3) with a higher yield (more than two-fold) and was less susceptible to precipitation than the WT protein. The crystal structure of TEM8 C177A that we solved later in this study revealed that residue 177 was buried in a hydrophobic core and isolated, similar to the corresponding CMG2 structure. Thus, it is reasonable to deduce that this single point mutation would not induce a large scale conformational change at the MIDAS site and related interface with PA. Hereafter, for simplicity, we refer to the TEM8 C177A single point mutant as WT.

### Crystallization

The TEM8 extracellular vWA domain crystals were grown by the hanging-drop vapor diffusion method at 22°C by mixing 1 µl of 5 mg/ml protein solution with 1 µl of reservoir solution (0.1 M sodium citrate trihydrate (pH 5.6), 0.2 M ammonium acetate, and 20% (w/v) polyethylene glycol 4000). We also directly added 0.1 M hexammine cobalt (III) chloride (Hampton Research) as an additive to the hanging drop (10% volume ratio) during optimization. The shape of the final crystals was laminary, and the crystals attained their maximum size (0.1×0.1×0.02 mm) after 10 d.

### Data collection and structure determination

Prior to data collection, the crystals were plunged into liquid nitrogen and transported to the cold nitrogen stream of beamline 17A at the Photon Factory synchrotron facility (Tsukuba, Japan). 20% (w/v) PEG 4000 in the reservoir solution was very suitable cryoprotectant for this crystal. Diffraction data were processed with the HKL2000 program at 1.7 Å resolution, and the crystal belongs to space group P1, with unit cell dimensions *a* = 65.9 Å, *b* = 66.1 Å, *c* = 74.4 Å, α = 63.7°, β = 88.2°, and γ = 59.9°. Our attempt to process the data in higher symmetry space groups failed because of significantly worse *R_merge_* values. The data collection statistics in the P1 crystal form are shown in Supplemental Data Table S1. Each asymmetric unit in the crystal contains six molecules of the TEM8 extracellular vWA domain. The diffraction phases were determined by the molecular replacement method, using the program PHASER [36] and the CMG2 vWA domain structure (PDB ID 1SHU) as the initial model. The TEM8 model was further built manually with COOT [37] and refined using REFMAC [38] from the CCP4 suite [39]. The TEM8 structure was refined to a final *R_free_* = 23.2% and *R_work_* = 19.4%. The stereochemistry was of excellent quality, as validated by the program PROCHECK [40]. Final refinement statistics are also summarized in Table S1.

### Protection of mammalian cells from PA intoxication

Murine monocyte–macrophage cells J774A.1 [American Type Culture Collection, Manassas, VA] were plated at a density of 30,000 cells/well in 96-well plates and cultured for 24 h before toxin treatment. A dilution series of TEM8 and its mutants, combined with PA proteins (100 ng/ml) and LF (100 ng/ml), was applied to the cells to a final volume of 100 µl/well. Cell viability was assayed 4 h after treatment by replacing the medium with 100 µl solution containing 1 mg/ml MTT (3-[4,5- dimethylthiazol-2-yl]-2,5-diphenyltetrazolium bromide), which was removed after a 1 h incubation at 37°C. The blue pigment (i.e., oxidized MTT) produced by viable cells was dissolved in 50 µl/well of 0.5% (w/v) SDS and 25 mM HCl in 90% (v/v) isopropanol, and the plates were vortexed. The A_570_ of oxidized MTT was measured using a Microplate Reader Model 550 (Bio-Rad Inc, Foster), and the data were analyzed with Prism software (GraphPad Software Inc, San Diego) as the percentage viability of control wells containing LF without PA. IC_50_ values were determined by nonlinear regression sigmoidal dose-response analysis with variable slopes. Each assay was performed in triplicate, and the assay was repeated at least four times independently. Data from representative assays are shown in Figure 4.

### pH-dependent conversion of the prepore to an SDS-resistant state

Receptor protein (15 µg) was added to PA_63_ heptamer prepore (25 µg). MgCl**_2_** was added to the reaction to a final concentration of 1 mM, and the mixture was left at room temperature for 20 min to allow complete binding. Reactions were divided into aliquots and incubated for 1 h with equal volumes of the following buffers: 1 M MES (pH 6.0), 1 M MES (pH 6.2), 1 M MES (pH 6.4), 1 M MES (pH 6.6), 1 M MES (pH 6.8), or 1 M HEPES (pH 7.0). Samples were then mixed with 2% SDS loading buffer of for 20 min. Their molecular weights were analyzed on a 3–12% Tris-Glycine gel in SDS running buffer. The protein complex bands were visualized by Coomassie Blue staining and digitalized with the computer program Bandscan (Glyko Inc).

### SPR assays

Surface plasmon resonance, to measure the binding between PA and receptor variants, was performed using the Biacore 2000 system. Monomeric PA83 was covalently linked to the carboxylated dextran matrix. It was diluted to 4 µM in sodium acetate buffer (pH 5.0), injected onto the activated surface at a flow rate of 5 µl/min for 65 min, and then blocked with ethanolamine. The vWA domains of the receptor variants (i.e., the analytes) were diluted to various concentrations (50–800 nM) in HEPES buffered saline (HBS, i.e. 10 mM HEPES (pH 7.4), 150 mM NaCl, and 0.005% (v/v) TWEEN-20) with additional 1 mM Mg2+, and serial injections were made at 20 µl/min at 25°C. After sample analysis, CM5 baselines were regenerated with 10 mM/L glycine HCl (pH 2.0) for 15 s and borate buffer (10 mM sodium tetraborate (pH 8.5) and 1 M NaCl) for 15 s.

## Supporting Information

Figure S1 The MIDAS site of one TEM8 vWA domain molecule was blocked by the adjacent molecule in the crystal cell. Protein molecules are shown in ribbon presentation. Only two adjacent TEM8 vWA domain molecules are shown and labeled as A, B in the crystal. The MIDAS site of A molecule (purple) is highlighted with a pink background. The MIDAS site of molecule A is blocked by molecule B (red) from above by steric hindrance. PA cannot interact with A's MIDAS site.
(2.90 MB TIF)

Figure S2 Sedimentation Velocity Data for TEM8. Curve represents a sedimentation velocity run for TEM8. This curve has only one peak (19.5 kD) that is close to the TEM8 vWA domain monomer's theoretic molecular weight, which demonstrates the TEM8 vWA domain molecule to exist as monomer in the solution. Sedimentation velocity (SV) experiment was conducted with an Optima XL-L analytical ultracentrifuge (Beckman-Coulter Instruments). An An60Ti rotor and standard six-sector equilibrium centerpieces were used. Freshly prepared TEM8 was further purified and buffer-exchanged into sedimentation buffer (150 mM NaCl and 25 mM Tris-HCl, pH 8.0) using a gel filtration column. c(M) distribution for TEM8 (1 mg/ml, Black line) obtained from sedimentation velocity experiments, at 20°C and a speed of 40,000 rpm. Absorbance scans were carried out at a wavelength of 280 nm, and 98 scans were collected at 2 min intervals.
(0.75 MB TIF)

Table S1 Data collection and refinement statistics.
(0.05 MB DOC)
