# Supplementary figures and images for: The Structure of Tumor Endothelial Marker 8 (TEM8) Extracellular Domain and Implications for Its Receptor Function for Recognizing Anthrax Toxin
Source: PLoS One. 2010 Jun 18;5(6):e11203. doi: 10.1371/journal.pone.0011203 (PMC2887854; doi:10.1371/journal.pone.0011203)

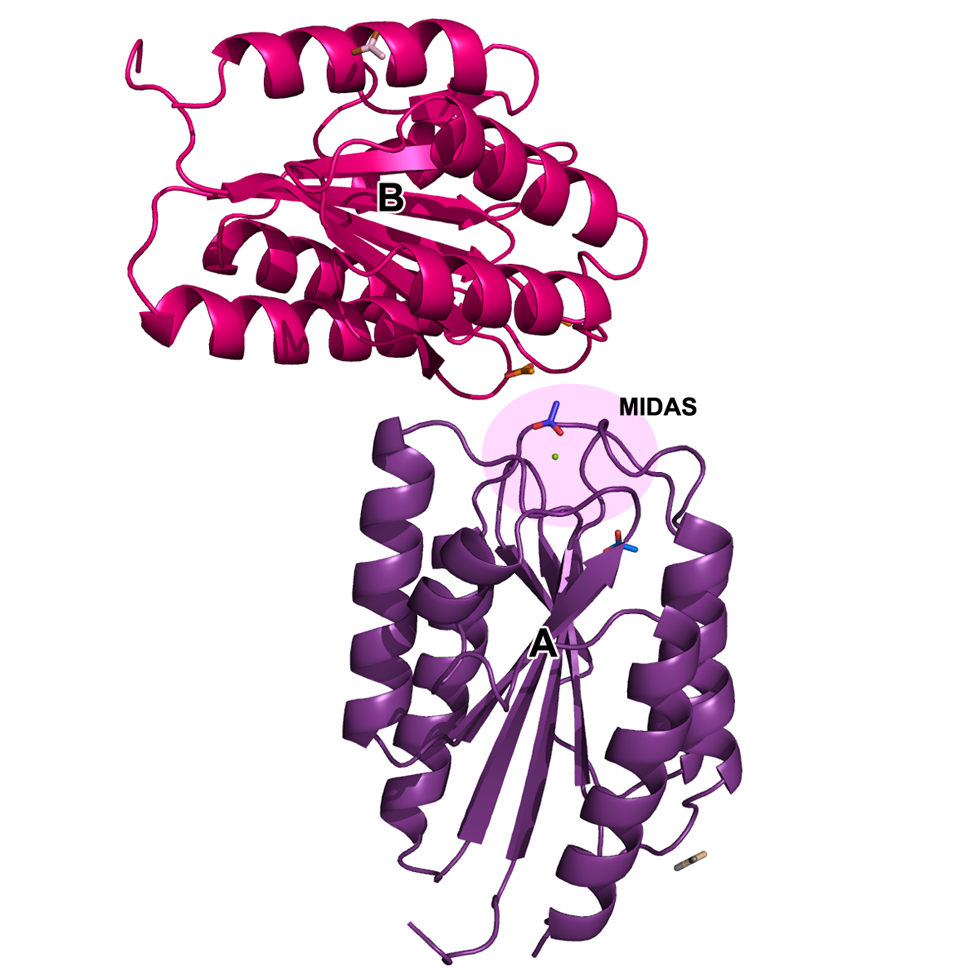

Supplement: Figure S1 — The MIDAS site of one TEM8 vWA domain molecule was blocked by the adjacent molecule in the crystal cell. Protein molecules are shown in ribbon presentation. Only two adjacent TEM8 vWA domain molecules are shown and labeled as A, B in the crystal. The MIDAS site of A molecule (purple) is highlighted with a pink background. The MIDAS site of molecule A is blocked by molecule B (red) from above by steric hindrance. PA cannot interact with A's MIDAS site. (2.90 MB TIF) [file pone.0011203.s001.tif]
