# Supplementary material for: The Structure of Tumor Endothelial Marker 8 (TEM8) Extracellular Domain and Implications for Its Receptor Function for Recognizing Anthrax Toxin
Source: PLoS One. 2010 Jun 18;5(6):e11203. doi: 10.1371/journal.pone.0011203 (PMC2887854; doi:10.1371/journal.pone.0011203)
Supplement: Table S1 — Data collection and refinement statistics. (0.05 MB DOC) [file pone.0011203.s003.doc]

Table S1. Data collection and refinement statistics

| | **Parameters** |  | | | --- | --- | --- | | **Data collection statistics** | | | | Cell parameters | *a*= 65.9 Å *, b*= 66.1Å, *c*= 74.4 Å  α= 63.7º, β= 88.2º, γ= 59.9º | | | Space group | *P1* | | | Wavelength used (Å) | 0.9798 | | | Resolution (Å) | 60 (1.76)^c^ – 1.70 | | | No. of reflections | 447,511 | | | No. of unique reflections | 91,416 | | | Completeness (%) | 87.9 (42.0) | | | Average I/σ(I) | 12.9 (1.4) | | | R_merge_^a^ (%) | 10.4 (39.5) | | | **Refinement statistics** | | | | R_work_^b^ (%) | 19.4 | | | R_free_^b^ (%) | 23.2 | | | r.m.s.d. bond distance (Å) | 0.006 | | | r.m.s.d. bond angle (º) | 0.890 | | | Ramachandran plot (excluding Pro & Gly) | |  | | Res. in most favored regions | | 155 (93.4 %) | | Res. in additionally allowed regions | | 9 (5.4%) | | Res. in generously allowed regions | | 2 (1.2 %) | | Res in disallowed regions | | 0 (0%) |   ^a^ *R_merge_* = Σ_h_Σ_l_ | I_ih_−<I_h_> |/Σ_h_Σ_I_ <I_h_>, where <I_h_> is the mean of the observations I_ih_ of reflection h.  ^b^ *R_work_* = Σ( ||F_p_(obs)|−|F_p_(calc)||)/ Σ|F_p_(obs)|; *R_free_* = R factor for a selected subset (5%) of the reflections that was excluded in prior refinement calculations.  ^c^ The numbers in parentheses are the corresponding values for the highest resolution shell.  ^d^ The Ramachandran plot was calculated with the program Procheck[1]. |
| --- | --- | --- | --- | --- | --- | --- | --- | --- | --- | --- | --- | --- | --- | --- | --- | --- | --- | --- | --- | --- | --- | --- | --- | --- | --- | --- | --- | --- | --- | --- | --- | --- | --- | --- | --- | --- | --- | --- | --- | --- | --- | --- | --- | --- | --- | --- | --- | --- | --- | --- | --- | --- | --- | --- | --- | --- | --- | --- | --- | --- | --- | --- | --- |

1. Laskowski, R.A., D.S. Moss, and J.M. Thornton, *Main-chain bond lengths and bond angles in protein structures.* Journal of Molecular Biology, 1993. **231**(4): p. 1049-67.
